# puma: a Bioconductor package for propagating uncertainty in microarray analysis

**DOI:** 10.1186/1471-2105-10-211

**Published:** 2009-07-09

**Authors:** Richard D Pearson, Xuejun Liu, Guido Sanguinetti, Marta Milo, Neil D Lawrence, Magnus Rattray

**Affiliations:** 1 School of Computer Science, University of Manchester, Oxford Road, Manchester, M13 9PL, UK; 2 Wellcome Trust Centre for Human Genetics, University of Oxford, Roosevelt Drive, Oxford, OX3 7BN, UK; 3 College of Information Science and Technology, Nanjing University of Aeronautics and Astronautics, 29 Yudao Street, Nanjing 210016, PR China; 4 Department of Computer Science, University of Sheffield, Regent Court 211 Portobello Street, Sheffield, S1 4DP, UK; 5 ChELSI Institute, Department of Chemical and Process Engineering, University of Sheffield, Mappin Street, Sheffield, S1 3JD, UK; 6 NIHR Cardiovascular Biomedical Research Unit, Sheffield Teaching Hospitals NHS Trust, Beech Hill Road, Sheffield, S10 2RX, UK

## Abstract

**Background:**

Most analyses of microarray data are based on point estimates of expression levels and ignore the uncertainty of such estimates. By determining uncertainties from Affymetrix GeneChip data and propagating these uncertainties to downstream analyses it has been shown that we can improve results of differential expression detection, principal component analysis and clustering. Previously, implementations of these uncertainty propagation methods have only been available as separate packages, written in different languages. Previous implementations have also suffered from being very costly to compute, and in the case of differential expression detection, have been limited in the experimental designs to which they can be applied.

**Results:**

*puma *is a Bioconductor package incorporating a suite of analysis methods for use on Affymetrix GeneChip data. *puma *extends the differential expression detection methods of previous work from the 2-class case to the multi-factorial case. *puma *can be used to automatically create design and contrast matrices for typical experimental designs, which can be used both within the package itself but also in other Bioconductor packages. The implementation of differential expression detection methods has been parallelised leading to significant decreases in processing time on a range of computer architectures. *puma *incorporates the first R implementation of an uncertainty propagation version of principal component analysis, and an implementation of a clustering method based on uncertainty propagation. All of these techniques are brought together in a single, easy-to-use package with clear, task-based documentation.

**Conclusion:**

For the first time, the *puma *package makes a suite of uncertainty propagation methods available to a general audience. These methods can be used to improve results from more traditional analyses of microarray data. *puma *also offers improvements in terms of scope and speed of execution over previously available methods. *puma *is recommended for anyone working with the Affymetrix GeneChip platform for gene expression analysis and can also be applied more generally.

## Background

The analysis of microarray experiments typically involves a number of stages. The first stage for analysis of Affymetrix GeneChip arrays is usually the application of a summarisation method such as MAS5.0 or RMA in order to obtain an expression level for each probeset on each array. Subsequent analyses then use these expression levels, for example to determine differentially expressed (DE) genes, or to find clusters of genes and/or conditions. Although there are a number of summarisation methods which can give accurate point estimates of expression levels, few can also provide any information about uncertainty in expression levels (such as standard errors). Even for methods that can provide uncertainty information, this is rarely used in subsequent analyses due to the lack of available methods for dealing with such information. A large amount of potentially valuable information is therefore lost. Recently, there has been a growing trend for disregarding the probe-to-probeset annotation provided by the array manufacturer in favour of so-called "remapped" data (e.g. [1]). With remapped data the number of probes in a probeset varies greatly, and hence making use of within-probeset uncertainty is likely to be of even greater benefit in this case. Here, we introduce the *puma *Bioconductor package which makes a suite of uncertainty propagation methods available to a general audience.

The multi-mgMOS method [2] uses Bayesian methods on Affymetrix GeneChip data to associate credibility intervals with expression levels. This was made available through the Bioconductor package *mmgmos*. The noise-propagation in principal component analysis (NPPCA) method [3] can propagate the expression level uncertainty to improve the results of principal component analysis (PCA). This method was made available as matlab code. The probability of positive log ratio (PPLR) method [4] can combine uncertainty information from replicated experiments in order to obtain point estimates and standard errors of the expression levels within each condition. These point estimates and standard errors can then be used to obtain a PPLR score for each probeset, which can then be used to rank probesets by probability of differential expression (DE) between two conditions. The PUMA-CLUST method [5] uses uncertainty propagation to improve results of a typical clustering analysis. PPLR and PUMA-CLUST were made available as separate R packages, but were not released through Bioconductor. The algorithmic details of multi-mgMOS, NPPCA, PPLR and PUMA-CLUST are explored more fully in the next section.

While many microarray studies are concerned with identifying genes that are differentially expressed between two levels of a single factor, for example between cancer and non-cancer patients, microarrays are also increasingly being used in more complex experimental designs where more than one factor is varied. This is often achieved with a factorial-designed experiment, where each combination of the levels of each factor is tested. As well as enabling a researcher to identify the effects of multiple factors in a single experiment, a factorial design also enables the study of the effect of interactions between different factors. The PPLR method is not directly applicable to such experiments.

Perhaps the most popular Bioconductor package for analysis of differential expression is *limma *[6]. *limma *requires the creation of a design matrix, and optionally also a contrast matrix. A search through the archives of the Bioconductor mailing list will reveal that one of the biggest difficulties users have is the creation of these matrices. Affymetrix users, however, will often have provided much of the information required in these matrices in the form of phenotype data using the *affy *package. As well as being confusing for inexperienced users, the manual creation of design and contrast matrices can also lead to human error. Although the methods incorporated in the *puma *package can often give improved results when compared to competing methods, this can come at the cost of increased computation time due to the parameter estimation methods involved. This is particularly the case with the variational EM algorithm used for combining the information from replicates in the PPLR method, which can take many hours to run for a typical analysis on a single machine. Many users, however, will have access to the processing power of multiple cores, either through access to a multi-node cluster, through the use of multiple machines on a local network, or simply through the use of multiple processors or a single processor with multiple cores on a single machine.

## Introduction to puma algorithms

### multi-mgMOS and probe-level measurement error

Affymetrix GeneChips use multiple probe-pairs called a probe-set to interrogate gene expression profiles. Each probe-pair contains a perfect match (PM) probe and a mismatch (MM) probe. The PM probe is designed to measure the specific hybridisation of the target and the MM probe measures the non-specific hybridisation associated with its corresponding PM probe. However, microarray experimental data show that the MM probe also measures the specific hybridisation signal in practice and the intensities of both PM and MM probes vary in probe-specific ways. This makes the identification of the true signal difficult. The probabilistic model multi-mgMOS [2] assumes the intensities of PM and MM probes for a probe-set both follow gamma distributions with parameters accounting for specific and non-specific hybridisation, and probe-specific effects. Let *y*_*ijc *_and *m*_*ijc *_represent the *j*th PM and MM intensities respectively for the *i*th probe-set under the *c*th condition. The model is defined by

(1)

where Ga represents the gamma distribution. The parameter *a*_*ic *_accounts for the background and non-specific hybridisation associated with the probe-set and *a*_*ic *_accounts for the specific hybridisation measured by the probe-set. The parameter *b*_*ij *_is a latent variable which models probe-specific effects.

The Maximum a Posteriori (MAP) solution of this model can be found by efficient numerical optimisation. The posterior distribution of the logged gene expression level can then be estimated from the model and approximated by a Gaussian distribution with a mean, , and a variance, *v*_*ic*_. The mean of this distribution is taken as the estimated gene expression for gene *i *under the condition *c *and the variance can be considered the measurement error associated with this estimate. The Gaussian approximation to the posterior distribution is useful for propagating the probe-level measurement error in subsequent downstream analyses.

### Including measurement uncertainty in finding DE genes

The PPLR method [4] includes probe-level measurement error in a hierarchical Bayesian model to detect differentially expressed (DE) genes. For a particular gene in PPLR, the observed logged expression level for the *i*th replicate under the *j*th condition is assumed to follow a Gaussian distribution,

(2)

where *μ*_*j *_is the mean logged expression level under condition *j*, *λ*_*j *_is the inverse of the between-replicates variance and v_*ij *_is the probe-level measurement error, which can be calculated from probabilistic probe-level analysis methods such as multi-mgMOS.

PPLR assumes that the parameters *θ *= {{*μ*_*j*_}, {*λ*_*j*_}} are independent and  is shared across different conditions to capture the gene-specific variability. The priors of the parameters are:

(3)

(4)

where *φ *= {*μ*_0_, *η*_0_, *α*, *β*} are hyperparameters. Inference in the PPLR model is carried out with a variational Expectation-Maximization (EM) algorithm. The estimated parameters are then used to calculate a PPLR score for finding DE genes.

#### Including measurement uncertainty in principal components analysis

We write the measurement error, *v*_*i*_, as a vector capturing the main technical sources of variance of the measured expression level on each chip *i*. PCA can be viewed as the maximum likelihood solution of a probabilistic factor analysis model [7] and [3] add the measurement error, *v*_*i*_, as an additional term in the observation noise of this model,

(5)

Unlike standard PCA there is no longer a closed form maximum likelihood solution and an iterative EM algorithm is used for parameter estimation.

#### Including measurement uncertainty in mixture clustering

Similarly to NPPCA, PUMA-CLUST [5] includes the measurement error of each data point in a standard Gaussian mixture model. Suppose *x*_*i *_is the true expression level for data point *i*. The *k*th component of the Gaussian mixture model is modelled by *p*(*x*_*i*_|*k*; *θ*_*k*_) =  (*x*_*i*_|*μ*_*k*_, Σ_*k*_). For the measured expression level  the *k*th Gaussian component can be augmented as

(6)

where diag(*v*_*i*_) represents the diagonal matrix whose diagonal entries starting in the upper left corner are the elements of *v*_*i*_.

This version of PUMA-CLUST treats each chip as an individual condition. For replicated data we have developed an improved method which propagates measurement error to a robust Student's t mixture model. Once published, this method will be incorporated into the puma package

## Contributions

The *puma *package combines the various methods described above in a single, easy-to-use package, and overcomes some of the shortcomings of these methods. *puma *offers the following contributions:

• pumaDE – an extension of the PPLR method to the multi-factorial case.

• The automated creation of design and contrast matrices for typical experimental designs.

• pumaComb – an implementation of the method of combining information from replicates [4] that is significantly speeded up through the use of parallel processing.

• pumaPCA – an R implementation of NPPCA, with much improved execution speed over the previous matlab version.

• Bringing together for the first time in a single package a suite of algorithms for propagating uncertainty in microarray analysis, together with tools for plotting, data manipulation, and comparison to other methods.

• Demonstration of uncertainty propagation methods on "remapped" Affymetrix GeneChip data.

## Implementation

*puma *is a Bioconductor package, and as such is free to obtain, is available on all common computer platforms, and is open source making the methods completely transparent to the end-user. Most of the core algorithms have been implemented in C code for speed, with the remainder of the package implemented in R. We have endeavoured to reuse as much existing Bioconductor code as possible, in particular the use of common classes for holding data. This enables easier comparison of our methods with other methods, and we encourage users to do this.

### Multi-factorial extension of PPLR

The calculation of PPLR between two conditions is given in equation (15) of [4]. In *puma *we have extended this to arbitrarily complex contrasts. For example, the interaction term between two 2-level factors can be calculated as:

(7)

where *P(...|D*) denotes the posterior probability after observing data *D*, and *μ*_*ij *_corresponds to the mean expression when the two factors take values *i *and *j*.

Under the variational approximation developed in [4], the mean of each condition has a Gaussian posterior distribution. Therefore the above integral is easily calculated.

### Automated creation of design and contrast matrices

The *puma *package has been designed to be as easy-to-use as possible for end users who have little experience with R and Bioconductor. One particularly important manifestation of this is the automated creation of design and contrast matrices. The details of this are included in the *puma *User Guide [8], but in essence the following contrasts are deemed as potentially interesting within *puma*:

• All pairwise comparisons within each factor.

• Comparisons of one level vs all other levels for factors with three or more levels.

• All main effects of factors.

• All interaction terms (up to three way) between factors.

### Parallelisation

We have parallelised the most time-consuming step of a typical *puma *analysis (running the function pumaComb) by making use of the R package *snow*. The use of *snow *means that the parallel processing can be carried out on a large number of different architectures including multi-core processors, multi-processor machines, clusters running various versions of MPI and heterogeneous networks running PVM.

### Using puma

multi-mgMOS [2] is implemented in the function mmgmos. The NPPCA method [3] is implemented in the function pumaPCA. The probability of positive log ratio (PPLR) method of [4] is implemented in the functions pumaComb (for combining information from replicates) and pumaDE (for determining differential expression from the combined information). PUMA-CLUST [5] is implemented in the function pumaClust. Each of these functions is described in separate sections in Results and Discussion.

We have implemented a separate Bioconductor experimental data package *pumadata *which contains example data sets that can help new users get up to speed with using *puma*. *puma *can be installed by first installing the latest version of R, and then running the following two commands from the R command line:

*> source("")*

*> biocLite("puma")*

Similarly, *pumadata *can be installed with the following command:

*> biocLite("pumadata")*

## Results and discussion

### Accounting for Uncertainty in Probeset Summarisation

The first step in a typical analysis is to load in data from Affymetrix CEL files, using the ReadAffy function from the *affy *package [9]. *puma *makes extensive use of phenotype data, which maps arrays to the condition or conditions of the biological samples from which the RNA hybridised to the array was extracted. It is recommended that this phenotype information is supplied at the time the CEL files are loaded. If the phenotype information is stored in the AffyBatch object in this way, it will then be made available for all further analyses. Details of how to include such phenotype information are included in the *puma *User Guide [8].

The recommended summarisation method to use within *puma *is multi-mgMOS [2]. The following code shows how to use this method on an example data set included in the *pumadata *package. We also create a summarisation using RMA [10] for comparison.

*> library(pumadata)*

*> data(affybatch.estrogen)*

*> eset_estrogen_mmgmos <- mmgmos(affybatch.estrogen)*

*> eset_estrogen_rma <- rma(affybatch.estrogen)*

mmgmos takes significantly longer to run than rma. The above commands took 225 and 4 seconds respectively to complete on a 2.93 GHz Intel Core 2 Duo MacBook Pro. multi-mgMOS performs well on the affycomp benchmark [11,12], giving the best score for 3 of the 14 measures (5. Signal detect slope, 6. low.slope and 9. Obs-intended-fc) on the HGU95 spike-in data set, and 1 of the 14 measures (10. Obs-(low)int-fc slope) on the HGU133 data set (data correct as of June 1, 2009).

Propagating Uncertainty in Principal Component Analysis

A useful first step in any microarray analysis is to look for gross differences between arrays. This can give an early indication of whether arrays are grouping according to the different factors being tested. This can also help to identify outlying arrays, which might indicate problems, and might lead an analyst to remove some arrays from further analysis. Principal components analysis (PCA) is often used for determining such gross differences. *puma *has a variant of PCA called Propagating Uncertainty in Microarray Analysis Principal Components Analysis (pumaPCA) which can make use of the uncertainty in the expression levels determined by multi-mgMOS. The following code shows what samples have been hyrbridised to each array, and then runs both pumaPCA and standard PCA (using prcomp) on the results obtained from the summarisation steps in the previous section. Following this, the 8 arrays used are plotted on the first two principal components using each method.

*> pData(eset_estrogen_mmgmos)*

      estrogen   time.h

low10-1.cel   absent   10

low10-2.cel   absent   10

high10-1.cel   present   10

high10-2.cel   present   10

low48-1.cel   absent   48

low48-2.cel   absent   48

high48-1.cel   present   48

high48-2.cel   present   48

*> pumapca_estrogen <- pumaPCA(eset_estrogen_mmgmos)*

*> pca_estrogen <- prcomp(t(exprs(eset_estrogen_rma)))*

*> par(mfrow = c(1, 2))*

*> plot(pumapca_estrogen, legend1pos = "right", legend2pos = "top",*

*+   main = "pumaPCA")*

*> plot(pca_estrogen$x, xlab = "Component 1", ylab = "Component 2",*

*+   pch = unclass(as.factor(pData(eset_estrogen_rma)[,*

*+      1])), col = unclass(as.factor(pData(eset_estrogen_rma)[,*

*+      2])), main = "Standard PCA")*

**Figure 1:**
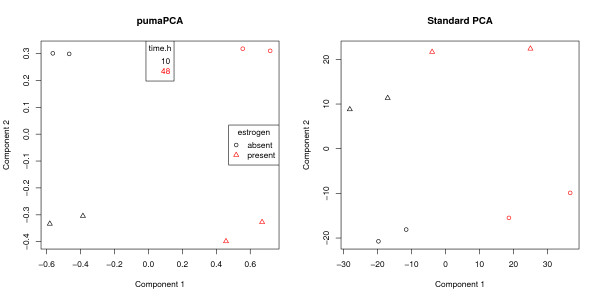
**Comparison of pumaPCA and standard PCA**. First two components after applying pumapca and prcomp to the estrogen data set processed by multi-mgMOS and RMA respectively.

pumaPCA is much more computationally demanding than standard PCA. The above pumaPCA and prcomp calls took 31 and 0.03 seconds respectively to complete on a 2.93 GHz Intel Core 2 Duo MacBook Pro. We can see from the phenotype data that this experiment has 2 factors (estrogen and time.h), each of which has two levels (absent vs present, and 10 vs 48), hence this is a 2 × 2 factorial experiment. For each combination of levels we have two replicates, making a total of 2 × 2 × 2 = 8 arrays. It can be seen from Figure 1 that the first component appears to be separating the arrays by time, whereas the second component appears to be separating the arrays by presence or absence of estrogen. Note that grouping of the replicates is much tighter with multi-mgMOS/pumaPCA. With RMA/PCA, one of the absent.48 arrays appears to be closer to one of the absent.10 arrays than to the other absent.48 array. This is not the case with multi-mgMOS/pumaPCA. We have seen similar patterns in other experiments (data not shown).

### Identifying differentially expressed genes

There are many different methods available for identifying differentially expressed (DE) genes. *puma *incorporates the Probability of Positive Log Ratio (PPLR) method [4]. The PPLR method can make use of the information about uncertainty in expression levels provided by multi-mgMOS. This proceeds in two stages. Firstly, the expression level information from the different replicates of each condition is combined using the function pumaComb to give a single expression level (and standard error of this expression level) for each condition. Following this, differentially expressed genes are determined using the function pumaDE. The following code determines DE genes from the estrogen data using multi-mgMOS/PPLR, and also, for comparison purposes, using RMA/limma.

*> eset_estrogen_comb <- pumaComb(eset_estrogen_mmgmos)*

*> pumaDERes <- pumaDE(eset_estrogen_comb)*

*> limmaRes <- calculateLimma(eset_estrogen_rma)*

**Figure 2:**
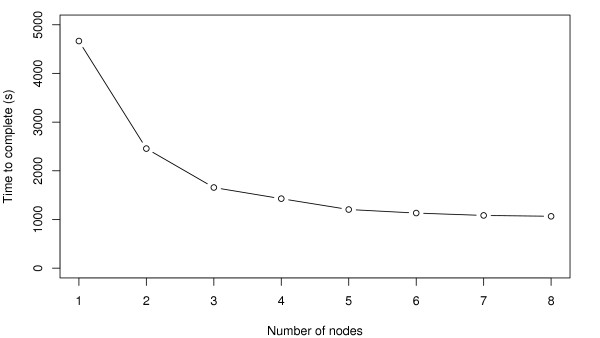
**Parallelisation speed-up**. Execution times for a typical run of the pumaComb function using different numbers of nodes.

Note that running the pumaComb command is typically the most time-consuming step in a typical *puma *analysis. As an example, the above command took 78 minutes to run on a 2.93 GHz Intel Core 2 Duo MacBook Pro. The computation time of this step can be decreased significantly when computed in parallel. Figure 2 shows typical run times when using different numbers of compute nodes on a Beowulf cluster. The pumaDE and calculateLimma commands each took less than a second to run.

Because this is a 2 × 2 factorial experiment, there are a number of contrasts that could potentially be of interest. *puma *will automatically calculate contrasts which are likely to be of interest for the particular design of a data set. For example, the following command shows which contrasts *puma *will calculate for this data set.

*> colnames(statistic(pumaDERes))*

[1] "present.10_vs_absent.10"

[2]"absent.48_vs_absent.10"

[3] "present.48_vs_present.10"

[4]"present.48_vs_absent.48"

[5] "estrogen_absent_vs_present"

[6] "time.h_10_vs_48"

[7] "Int__estrogen_absent.present_vs_time.h_10.48"

Here we can see that there are seven contrasts of potential interest. The first four are simple comparisons of two conditions. The next two are comparisons between the two levels of one of the factors. These are often referred to as "main effects". The final contrast is known as an "interaction effect". In more simple cases, where there are just two conditions, *puma *will create just one contrast.

Suppose we are particularly interested in the interaction term. We saw above that this was the seventh contrast identified by *puma*. The following commands will identify the gene deemed to be most likely to be differentially expressed due to the interaction term by the RMA/limma approach. We then plot the expression levels of this gene in the four conditions as determined by RMA and multi-mgMOS.

*> topLimmaIntGene <- topGenes(limmaRes, contrast = 7)*

*> par(mfrow = c(1, 2))*

*> plotErrorBars(eset_estrogen_rma, topLimmaIntGene)*

*> plotErrorBars(eset_estrogen_mmgmos, topLimmaIntGene)*

**Figure 3:**
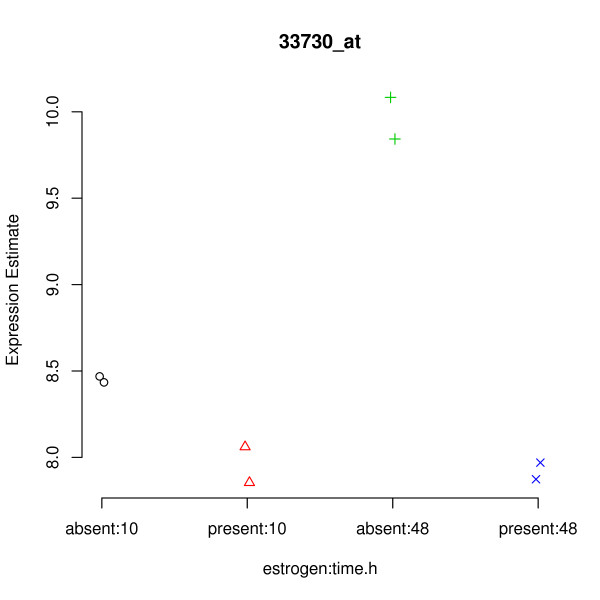
**Example of an apparently DE gene identified using RMA/limma**. RMA expression levels for the gene determined by RMA/limma to be most likely to be differentially expressed due to the interaction term in the estrogen data set.

**Figure 4:**
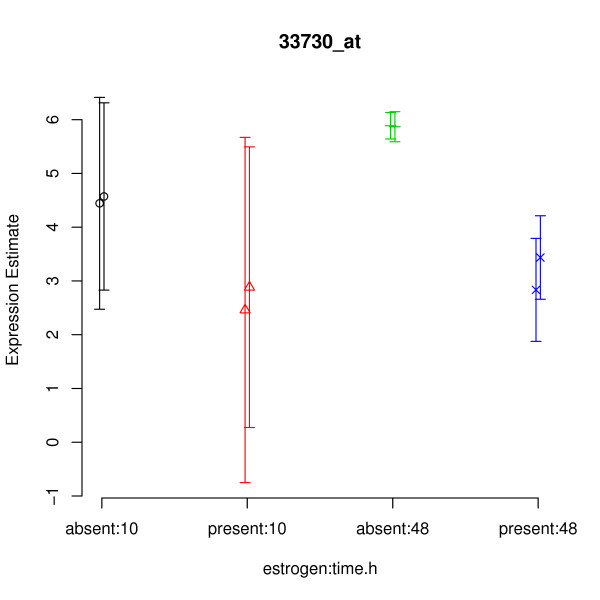
**Casting doubt on the example gene identified as DE using RMA/limma**. multi-mgMOS expression levels for the gene determined by RMA/limma to be most likely to be differentially expressed due to the interaction term in the estrogen data set. Note that multi-mgMOS provides error bars as well as point estimates for the expression levels.

The gene shown in Figure 3 would appear to be a good candidate for a DE gene. There seems to be an increase in the expression of this gene due to the combination of the estrogen = absent and time = 48 conditions. The within condition variance (i.e. between replicates) appears to be low, so it would seem that the effect we are seeing is real. The plot of Figure 4 tells a somewhat different story. Again, we see that the expected expression level for the absent:48 condition is higher than for other conditions. Also, we again see that the within condition variance of expected expression level is low (the two replicates within each condition have roughly the same value). However, we can now see that we actually have very little confidence in the expression level estimates (the error bars are large), particularly for the time = 10 arrays. Indeed the error bars of absent:10 and present:10 both overlap with those of absent:48, indicating that the effect previously seen might actually be an artifact.

The following code determines and plots the gene most likely to be differentially expressed due to the interaction term using multi-mgMOS and pumaDE. This analysis was not possible using previous implementations of multi-mgMOS and PPLR, as the PPLR method was only able to determine differential expression between two levels of a single condition.

*> toppumaDEIntGene <- topGenes(pumaDERes, contrast = 7)*

*> plotErrorBars(eset_estrogen_mmgmos, toppumaDEIntGene)*

**Figure 5:**
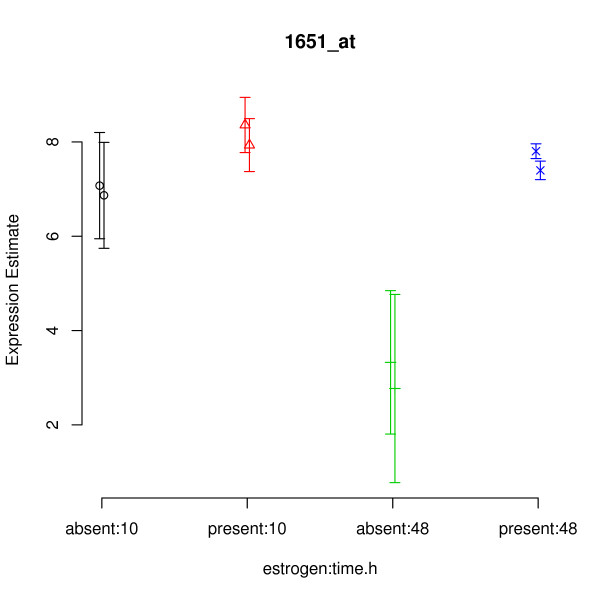
**Example showing benefits of using multi-mgMOS/PPLR for differential expression detection**. Expression levels and error bars (as calculated by multi-mgMOS) for the gene determined most likely to be differentially expressed due to the interaction term in the estrogen data set by mmgmos/pumaDE.

Figure 5 shows the gene determined by multi-mgMOS/PPLR to be most likely to be differentially expressed due to the interaction term. There appears to be lower expression of this gene due to the combination of the estrogen = absent and time = 48 conditions. Unlike with the gene shown in the plot of Figure 4 there is no overlap in the error bars between this condition, and the other conditions. Hence, this would appear to be a better candidate for a gene differentially expressed due to the interaction term. The combination of multi-mgMOS and PPLR (as implemented in the functions mmgmos and pumaComb/pumaDE) gave the strongest performance amongst 42 combinations of summarisation and DE detection methods in version 1.1 of the AffyDEComp benchmark [13].

### Clustering with pumaClust

The following code will identify seven clusters from the output of mmgmos:

*> pumaClust_estrogen <- pumaClust(eset_estrogen_mmgmos,*

*+   clusters = 7)*

Clustering is performing .....................................................................

Done.

The result of this is a list with different components such as the cluster each probeset is assigned to and cluster centers. The following code will identify the number of probesets in each cluster, the cluster centers, and will write out a csv file with probeset to cluster mappings:

*> summary(as.factor(pumaClust_estrogen$cluster))*

*> matplot(t(pumaClust_estrogen$centers))*

*> write.csv(pumaClust_estrogen$cluster, file = "pumaClust_clusters.csv")*

Examples of improved performance on real and simulated data sets of PUMA-CLUST when compared with a standard Gaussian mixture model (MCLUST) are given in [5]

### Analysis using remapped CDFs

There is increasing awareness that the original probe-to-probeset mappings provided by Affymetrix are unreliable for various reasons. Various groups have developed alternative probe-to-probeset mappings, or "remapped CDFs", and many of these are available either as Bioconductor annotation packages, or as easily downloadable cdf packages. One of the issues with using remapped CDFs is that many probesets in the remapped data have very few probes. This makes reliable estimation of the expression level of such probesets even more problematic than with the original mappings. Because of this, we believe that even greater attention should be given to the uncertainty in expression level measurements when using remapped CDFs than when using the original mappings. In the *puma *User Guide [8], we give an example of using a remapped CDF package created using AffyProbeMiner [1]. We show that, as with the standard Affymetrix annotation, we can improve results of both PCA and DE detection using *puma *methods on the remapped data.

### Application beyond Affymetrix microarray data

Although the methods within *puma *were originally designed for use with Affymetrix microarray expression data, there is considerable scope for application beyond this domain. This is particularly so for the functions pumaComb, pumaDE, pumaPCA and pumaClust. pumaComb and pumaDE can be used in situations where the probability of a difference between means is required from data which has associated standard errors. One directly related application is the analysis of Illumina BeadArray data. Rather than using multi-mgMOS to determine standard errors of expression levels as is recommended with Affymetrix data, the empirical standard errors output by Illumina's BeadStudio software, or the Bioconductor package *beadarray *[14] can be used directly with pumaComb and pumaDE to determine differentially expressed genes. More generally, pumaComb and pumaDE can be used as an alternative to a t-test to determine probabilities of differences between means of data from different classes, where those data have both point estimates and standard errors associated with those estimates. Similarly, pumaPCA and pumaClust can be applied more generally as alternatives to methods such as standard PCA and standard clustering algorithms respectively.

## Conclusion

The *puma *package makes use of uncertainty propagation to give improved performance when compared to more traditional methods of differential expression detection, principal component analysis and clustering. The package can be used for analysis of Affymetrix GeneChip data, but can also be applied more generally. The package extends previous work by extending the PPLR method to the multi-factorial case, and by implementing the NPPCA algorithm for the first time in R. The package also incorporates a large number of features which make anlaysis easier and quicker to run, including parallelisation of the pumaComb function, automated creation of design and contrast matrices, and tools for plotting, data manipulation, and comparison to other methods. *puma *is available freely from Bioconductor.

### Availability and requirements

• Project name: puma

• Project homepage: 

• Operating systems: Platform independent

• Programming language: R, C

• Other requirements: R

• License: LGPL except puma uses donlp [15] which has the following conditions of use:

1. donlp2 is under the exclusive copyright of P. Spellucci (e-mail:spellucci@mathematik.tu-darmstadt.de) "donlp2" is a reserved name

2. donlp2 and its constituent parts come with no warranty, whether expressed or implied, that it is free of errors or suitable for any specific purpose. It must not be used to solve any problem, whose incorrect solution could result in injury to a person, institution or property. It is at the users own risk to use donlp2 or parts of it and the author disclaims all liability for such use.

3. donlp2 is distributed "as is". In particular, no maintenance, support or trouble-shooting or subsequent upgrade is implied.

4. The use of donlp2 must be acknowledged, in any publication which contains results obtained with it or parts of it. Citation of the authors name and netlib-source is suitable.

5. The free use of donlp2 and parts of it is restricted for research purposes. Commercial uses require permission and licensing from P. Spellucci.

## List of abbreviations

mgMOS: modified gamma Model Of Signal; multi-mgMOS: multi-chip modified gamma Model Of Signal; NPPCA: noise-propagation in principal component analysis; PPLR: probability of positive log ratio; DE: differentially expressed.

## Authors' contributions

RDP extended the PPLR method to factorial experiments, developed the puma package from earlier code, maintains puma, and devised and wrote the manuscript. XL originally developed the mmgMOS and PPLR methods. GS partly developed the original matlab code for NPPCA. MM developed the code for mgMOS. NDL partly developed the original matlab code for NPPCA and partly initiated the puma project. MR partly initiated the puma project and supervised the development of the puma package.
